# LPS-treatment of bovine endometrial epithelial cells causes differential DNA methylation of genes associated with inflammation and endometrial function

**DOI:** 10.1186/s12864-020-06777-7

**Published:** 2020-06-03

**Authors:** Naveed Jhamat, Adnan Niazi, Yongzhi Guo, Metasu Chanrot, Elena Ivanova, Gavin Kelsey, Erik Bongcam-Rudloff, Göran Andersson, Patrice Humblot

**Affiliations:** 1 Department of Animal Breeding and Genetics, Section of Molecular Genetics, Swedish University of Agricultural Sciences, Uppsala, 750 07 Sweden; 2 SLU-Global Bioinformatics Centre, Swedish University of Agricultural Sciences, Uppsala, 750 07 Sweden; 3 Department of Information Technology, University of the Punjab, Gujranwala Campus, Gujranwala, Pakistan; 4 Department of Clinical Sciences, Division of Reproduction, Swedish University of Agricultural Sciences, Uppsala, 750 07 Sweden; 5 Faculty of Veterinary Science, Rajamangala University of Technology Srivijaya, Nakhon Si Thammarat, 802 40 Thailand; 6 Epigenetics Programme, The Babraham Institute, Cambridge, UK; 7 Centre for Trophoblast Research, University of Cambridge, Cambridge, UK; 8 Centre for Reproductive Biology in Uppsala, CRU, P.O. Box 7054, 750 07 Uppsala, Sweden

**Keywords:** LPS, Bovine endometrial epithelial cells, RRBS, Endometritis, Inflammation, Implantation

## Abstract

**Background:**

Lipopolysaccharide (LPS) endotoxin stimulates pro-inflammatory pathways and is a key player in the pathological mechanisms involved in the development of endometritis. This study aimed to investigate LPS-induced DNA methylation changes in bovine endometrial epithelial cells (bEECs), which may affect endometrial function. Following in vitro culture, bEECs from three cows were either untreated (0) or exposed to 2 and 8 μg/mL LPS for 24 h.

**Results:**

DNA samples extracted at 0 h and 24 h were sequenced using reduced representation bisulfite sequencing (RRBS). When comparing DNA methylation results at 24 h to time 0 h, a larger proportion of hypomethylated regions were identified in the LPS-treated groups, whereas the trend was opposite in controls. When comparing LPS groups to controls at 24 h, a total of 1291 differentially methylated regions (DMRs) were identified (55% hypomethylated and 45% hypermethylated). Integration of DNA methylation data obtained here with our previously published gene expression data obtained from the same samples showed a negative correlation (*r* = − 0.41 for gene promoter, *r* = − 0.22 for gene body regions, *p* < 0.05). Differential methylation analysis revealed that effects of LPS treatment were associated with methylation changes for genes involved in regulation of immune and inflammatory responses, cell adhesion, and external stimuli. Gene ontology and pathway analyses showed that most of the differentially methylated genes (DMGs) were associated with cell proliferation and apoptotic processes; and pathways such as calcium-, oxytocin- and MAPK-signaling pathways with recognized roles in innate immunity. Several DMGs were related to systemic inflammation and tissue re-modelling including *HDAC4, IRAK1, AKT1, MAP3K6, Wnt7A* and *ADAMTS17.*

**Conclusions:**

The present results show that LPS altered the DNA methylation patterns of bovine endometrial epithelial cells. This information, combined with our previously reported changes in gene expression related to endometrial function, confirm that LPS activates pro-inflammatory mechanisms leading to perturbed immune balance and cell adhesion processes in the endometrium.

## Background

Endometritis is a common disease in post-partum dairy cows with negative impacts on reproductive performance and increased risk of culling, thus, causing major economic losses to the dairy industry [1, 2]. In case of infection by Gram negative bacteria such as *E. coli*, these effects are mediated by the lipopolysaccharide (LPS) endotoxin. LPS has been reported to impair reproductive performance in cattle [3] and affect early pregnancy in ewes [4]. The Toll-like receptor (TLR) signaling pathway is a central component of the primary innate immune response to pathogenic challenge. LPS stimulates the host’s innate immune response by increasing TLR4 and MyD88-dependent signaling [5, 6] and subsequently activates the expression of pro-inflammatory cytokines and chemokines, such as interleukin 1A (IL-1A), IL-6 and IL-8 [5–8] and activation of JAK / STAT signaling pathway [7]. These pathways are pivotal for host defense against pathogens during endometritis [7].

Appropriate balance in production of cytokines and growth factors in endometrial cells is important for embryo development and successful implantation. During endometritis, these processes may indirectly be compromised due to elevated levels of cytokines, affecting endometrial receptivity and subsequently perturbing critical embryo-maternal interactions [9, 10].

Epigenetic modifications, such as DNA methylation, have been shown to be associated with changes in gene expression in the endometrium during early pregnancy [11], and may regulate the uterine response to embryo implantation [12, 13]. Several studies on gene expression and DNA methylation mainly performed in the human species have highlighted important genes and pathways affecting reproductive function during early or late pregnancy stages [14–19]. Other studies addressed the impact of infection and LPS on the DNA methylation status of immune cells. In human macrophages, LPS induced specific methylation changes lead to inactivation of pro-inflammatory pathways [20].

Endometrial epithelial cells (EECs) are key players in the defense of the uterus against most inflammatory diseases by triggering immune responses [21–23]. During endometritis and at early stages of pregnancy, gene expression changes related to pathways including cell adhesion, cytoskeleton remodeling and cell proliferation were reported [24–26]. Despite this, in the cow, the information related to epigenetic regulation of the above pathways and of the immune response in EECs in case of uterine infection is scarce, to the best of our knowledge. DNA demethylation in bovine endometrial cells was observed after 24 h of LPS exposure in specific sites of IL-6 and IL-8 promoter regions [27]. However, genome-wide epigenetic approaches have not been used so far to investigate changes in DNA methylation of the bovine endometrial epithelial cells (bEECs) in response to molecules from pathogens and there is a lack of information on the epigenetic mechanisms induced by infection, which may contribute to alterations of endometrial function.

The aim of the present study was to identify genomic regions presenting differential DNA methylation in bEECs following exposure to LPS, by using reduced representation bisulfite sequencing (RRBS). We also investigated here how DNA methylation changes are correlated with the transcriptomic response to LPS from RNAseq data obtained from the same cell samples and treatment conditions [28]. Thus, providing insights in alterations of DNA methylation induced by LPS possibly influencing endometrial function.

## Results

### RRBS and DNA methylation profile

DNA methylation profiles were established from a set of 12 samples of post primary bEECs; three untreated samples at time 0 h and nine samples at 24 h after exposure to LPS (0, 2, 8 μg/mL; Additional file 1: Figure S1). These concentrations of LPS may mimic those previously reported in cow uterine fluid following cases of clinical endometritis and/or in vivo experimental infection [29, 30]. They were chosen here also, due to different phenotypic responses to LPS in terms of cell survival and proliferation profiles and proteomic profiles [31, 32]. For the sake of consistency when studying correlation between DNA methylation and gene expression results, the same biological material was used (same cells exposed to same LPS dosages and time point) as in our former RNAseq study [28]. Overall, RRBS yielded a total of 17–21 million reads per sample. After quality filtering, 60–62% of the reads were successfully aligned to the bovine reference genome sequence (bosTau8), whereas 40–50% of the reads were uniquely mapped. In total, we identified 2.1–2.3 million CpG sites per sample, of which 1.93 million were covered in all samples, representing 7.1% of the total number of CpGs (~ 27 M) in the *Bos taurus* genome. Raw sequencing data and mapping statistics are summarized in Additional file 2: Table S1.

**Fig. 1:**
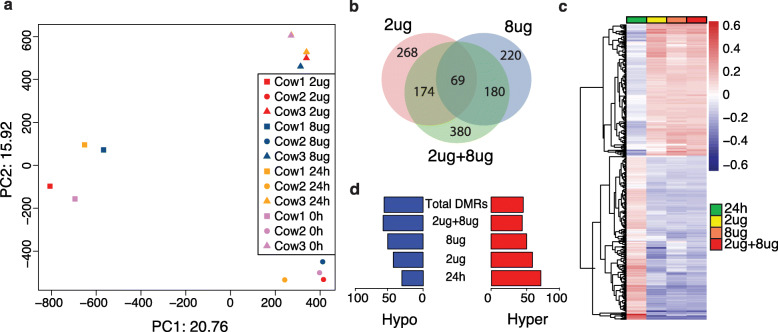
LPS effects on DNA methylation in bovine endometrial epithelial cells (bEECs). **a** Principal component analysis displaying overall methylation profiles across all samples. The first dimension explained 21% variation and separated Cow1 from Cow2 and Cow3. The second dimension explained 16% variation, separated both Cow2 versus Cow3. **b** Venn diagram displaying overlapping differentially methylated regions (DMRs) from 24 h sample groups: 0 μg vs. 2 μg (pink), 0 μg vs. 8 μg (blue), and 0 μg vs. 2 μg + 8 μg (green). **c** Heatmap of significant DMRs (1291) showing similar methylation trend for the analyses performed in (**b**). The scale shows hypermethylated (red) and hypomethylated (blue) levels for each DMR. **d** Bar plot showing distribution of the percent of hyper and hypomethylated DMRs when comparing time 0 h and 24 h in controls, and 24 h control with 2 μg or 8 μg, and 2 μg + 8 μg combined LPS groups. Top bar shows a similar pattern for total DMRs identified in 2 μg, 8 μg, and 2 μg + 8 μg analysis

From the RRBS data, differentially methylated regions (DMRs) throughout the bovine genome in response to LPS treatment were identified. CpG site coverage distribution showed that a large number of CpG sites had coverage of 10 reads or below in all samples (Additional file 1: Figure S2). A total of 700,323 CpG regions with at least one CpG site and read coverage ≥5 in all samples were obtained after tiling the genome for 100 bp regions. From those, 157,202 regions that contained ≥2 CpG sites were used for differential methylation analysis. Principal component analysis separated the samples according to individuals and did not reveal a strong effect of LPS on the DNA methylation pattern. This was related to the high degree of correlation between methylation profiles of treated and untreated groups (Fig. 1a; Additional file 1: Figure S3). However, differential DNA methylation analyses detected 511 and 469 significant DMRs (*q*-value < 0.05) in LPS-2 μg and LPS-8 μg, respectively, when compared to 24 h untreated control samples. The comparison between 0 h and 24 h control groups detected 822 DMRs of which 30% were hypomethylated and 70% were hypermethylated (Additional file 2: Table S2). We noted that a relatively low number of DMRs were shared between the 2 μg and 8 μg LPS groups when compared to 24 h control (Fig. 1b). In an attempt to recover DMRs that might be discarded due to coverage threshold, we combined data from the 2 and 8 μg LPS samples and compared them to 24 h control samples. The combined-LPS analysis detected 803 DMRs, sharing many DMRs identified in the two LPS groups (Fig. 1b). Finally, to avoid omission of functionally important methylated regions, we included in the analysis, those DMRs that did not withstand the *q*-value threshold in combined-LPS comparison but were significantly differentially methylated in either 2 μg or 8 μg LPS-treated samples. A total of 1291 DMRs were then identified and used for further analysis. From those, 707 (55%) were hypomethylated and 584 (45%) hypermethylated (Additional file 2: Table S3). The effect of LPS on the bEECs methylome showed similar methylation patterns in all treated groups (Fig. 1c). LPS treatment induced a higher proportion of hypomethylation when compared to control DMRs identified in the comparison between time 24 h and 0 h in controls (Fig. 1d).

### Genomic distribution of DMRs

**Fig. 2:**
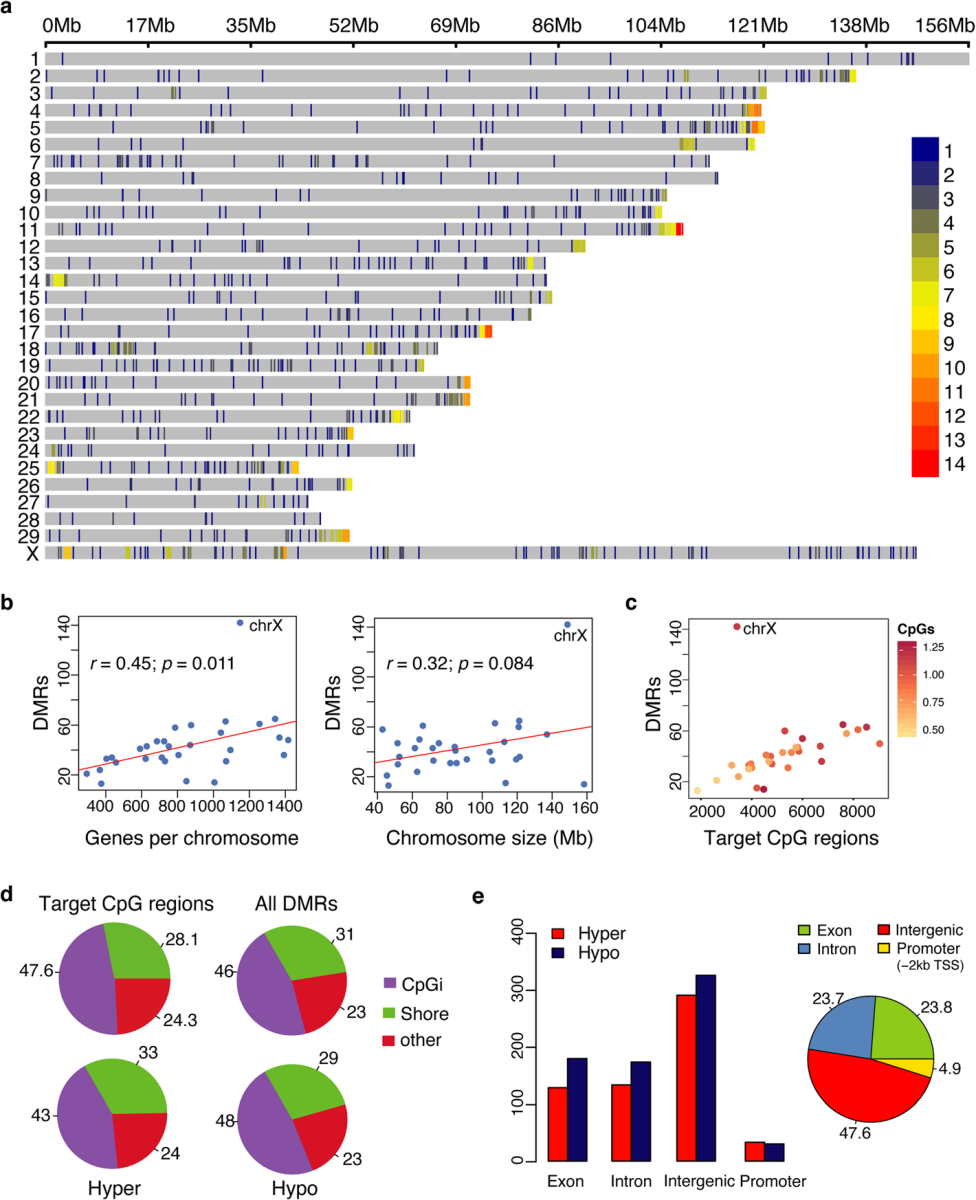
Genomic distribution of differentially methylated regions (DMRs). **a** Distribution of significant DMRs on 30 chromosomes of *Bos taurus*. Horizontal axis displays the chromosome length; 1–14 legend insert indicates the DMR density within 1 Mbp window size. **b** Scatterplots showing correlation of DMRs with number of genes per chromosome (left) and size of chromosomes (right). The Pearson’s correlation coefficients are shown on each plot. **c** Scatterplot showing distribution of DMRs against all targeted CpG regions (100 bp) on each chromosome. Colour intensity shows CG dinucleotide occurrence (million as unit) in the chromosomes. **d** Pie chart shows percentages of DMRs location in CpG islands, shores and other genomic regions. **e** Bar plot and pie chart shows distribution of DMRs in genic and non-genic regions. Exons and introns have annotation precedence over promoter regions, which are downstream (2 kb) of transcription-start sites (TSSs). For promoters, only DMRs 2 kb upstream of TSSs are shown

The chromosomal distribution of the DMRs was determined to assess whether or not DMRs were associated to specific chromosomal features. The distribution of DMRs was skewed towards chromosomal ends (Fig. 2a). The distribution of total targeted regions (*n* = 157,202) was not associated with telomeric regions (20 kb) of the chromosomes. On the contrary, sub-telomeric regions (within 2 Mb to telomeres) were significantly enriched for DMRs compared to non-telomeric regions (*Fisher’s Exact*, *p* < 1.14e-05). In addition, associations of DMRs with the number of genes per chromosome and size of chromosomes were tested (Fig. 2b). A significant positive correlation was found between the number of DMRs and the number of genes per chromosome (*r* = 0.45, *p* = 0.011). However, no significant correlation was noted with chromosomal size (*r* = 0.32, *p* = 0.084). Interestingly, 143 DMRs were detected on the X chromosome, which is twice as many compared to the average number of DMRs located on the autosomal chromosomes. This effect on gross differences in DMRs on the X chromosome compared with other chromosomes was independent of CpG richness of all chromosomes and targeted regions (Fig. 2c). When analyzing the distribution of DMRs in relation to genes and CpG islands 46% of the total number of identified DMRs were located in CpG islands, 31% at the shores, while 23% were located in other genomic regions (Fig. 2d), which corresponds to enrichment of these regions when using RRBS. A similar proportion of DMRs was located in intergenic regions (47.6%; *n* = 615) and within genes (47.5%; *n* = 613), whereas 4.8% (*n* = 63) were located in promoter regions (2 kb 5′ of the transcription start site) (Fig. 2e). The 600 differentially methylated genes (DMGs) having one or more DMRs included 589 protein-coding genes, seven miRNA, and four pseudogenes (Additional file 2: Table S4). Among genes that contained at least three DMRs in the gene body and promoter regions, *NSG1* had the highest number with five DMRs; four DMRs were found in *FAM19A5, SARDH* and *ENSBTAG00000046364*, while genes containing three DMRs were *PTMA*, *SLC20A2*, *IRAK1*, *PCDHGC3*, *HDAC4*, *VIPR2*, *C9orf172* (*AJM1*), and *ENSBTAG00000008542*.

### Correlation between DNA methylation and gene expression

**Fig. 3:**
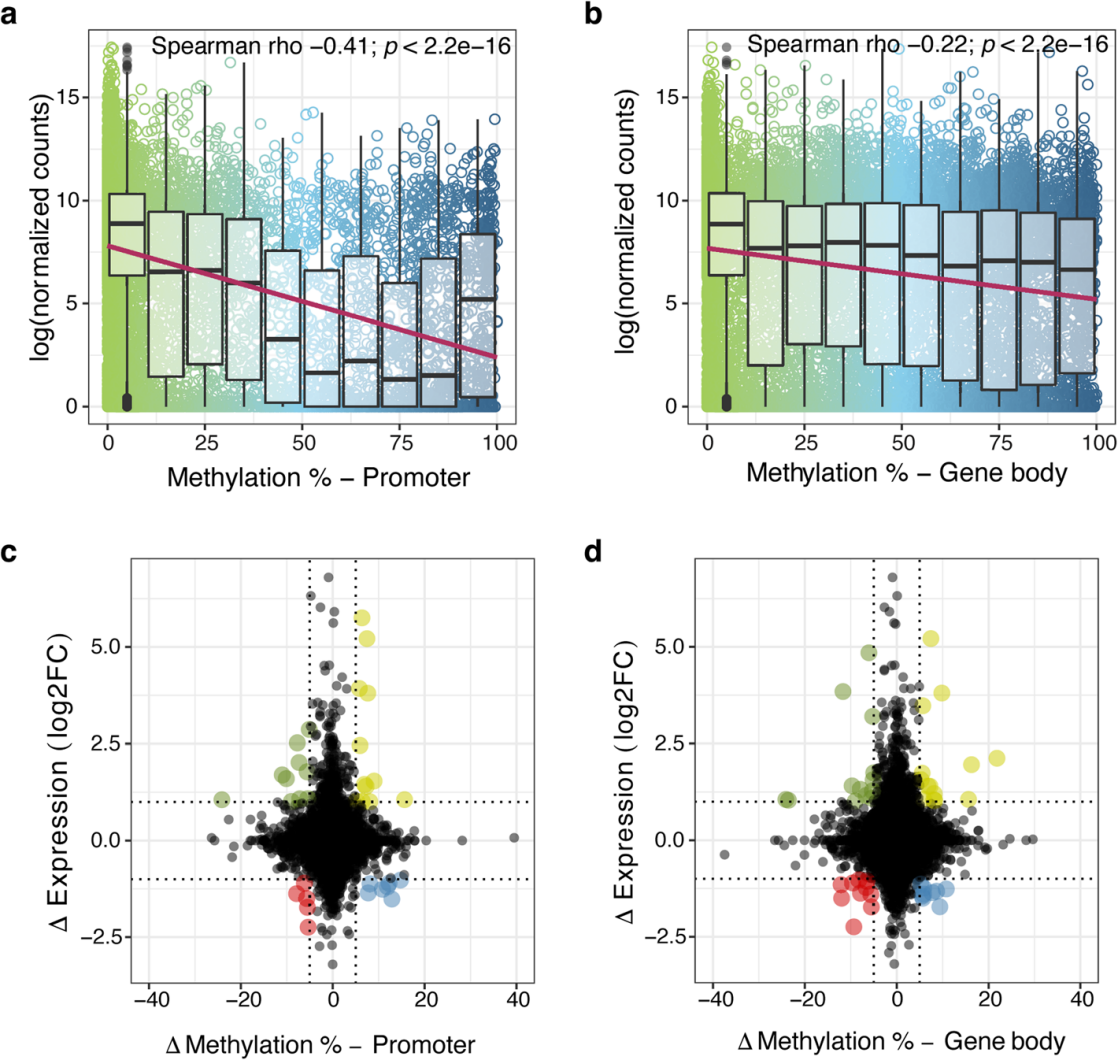
Integration of gene expression and methylome data. **a** Scatterplot showing mean gene expression and boxplot showing mean DNA methylation in differentially methylated regions (DMRs) in treated group for DMRs in promoters (**a**) and DMRs in gene bodies (**b**), with lines representing a linear trend. Bars in the box plot correspond to the median. The lower and upper hinges correspond to the first and third quartiles (the 25th and 75th percentiles). The lower/upper whisker extends from the hinge to the smallest/largest value no further than 1.5 * IQR (inter quartile range) from the hinges. **c**–**d** Scatterplots displaying the effect of lipopolysaccharide (LPS) on the transcriptome and the methylome when compared to the control group; change in gene expression (log_2_ Fold Change) is plotted against change in DNA methylation for **c** promoters of 12,115 genes and (**d**) gene bodies of 13,263 genes. Highlighted points denote genes with |ΔMethylation| > 5% and | Δexpression log_2_FC| > 1; hypermethylated/increased expression (yellow), hypermethylated/lower expression (blue), hypomethylated/increased expression (green) and hypomethylated/lower expression (red)

The potential effect of DNA methylation on gene expression was characterized by comparing methylation and RNA expression data previously obtained by RNAseq on the same cell samples [28]. The transcriptome-wide association between gene expression and DNA methylation within promoter regions and gene bodies was examined. There was a significant negative association between mean methylation of promoter regions and gene expression (Spearman rho = − 0.41; *p <* 2.2e-16; Fig. 3a). Although weaker, a significant negative relationship was also observed between gene body methylation and gene expression (Spearman rho = − 0.22, *p <* 2.2e-16; Fig. 3b).

In a further step, 80 genes for which there could be a functionally important effect of LPS on both DNA methylation and gene expression (|Δmethylation| > 5% and |Δexpression log_2_FC| > 1) were identified. For both promoters and gene body regions, there was no evidence for unequal distribution of genes (χ2 = 3.4, *p* = 0.33 and χ2 = 1.47, *p* = 0.68, respectively). However, 39 genes (49.9%) showed an inverse relationship between their degree of DNA methylation (in promoter or body) and gene expression (Fig. 3c, d; Additional file 2: Table S5). A combined functional analysis focusing only on the genes showing an inverse relationship (above threshold) revealed that these were related to ion/calcium ion transport and signal transduction processes.

### Gene ontology and pathway analyses

**Fig. 4:**
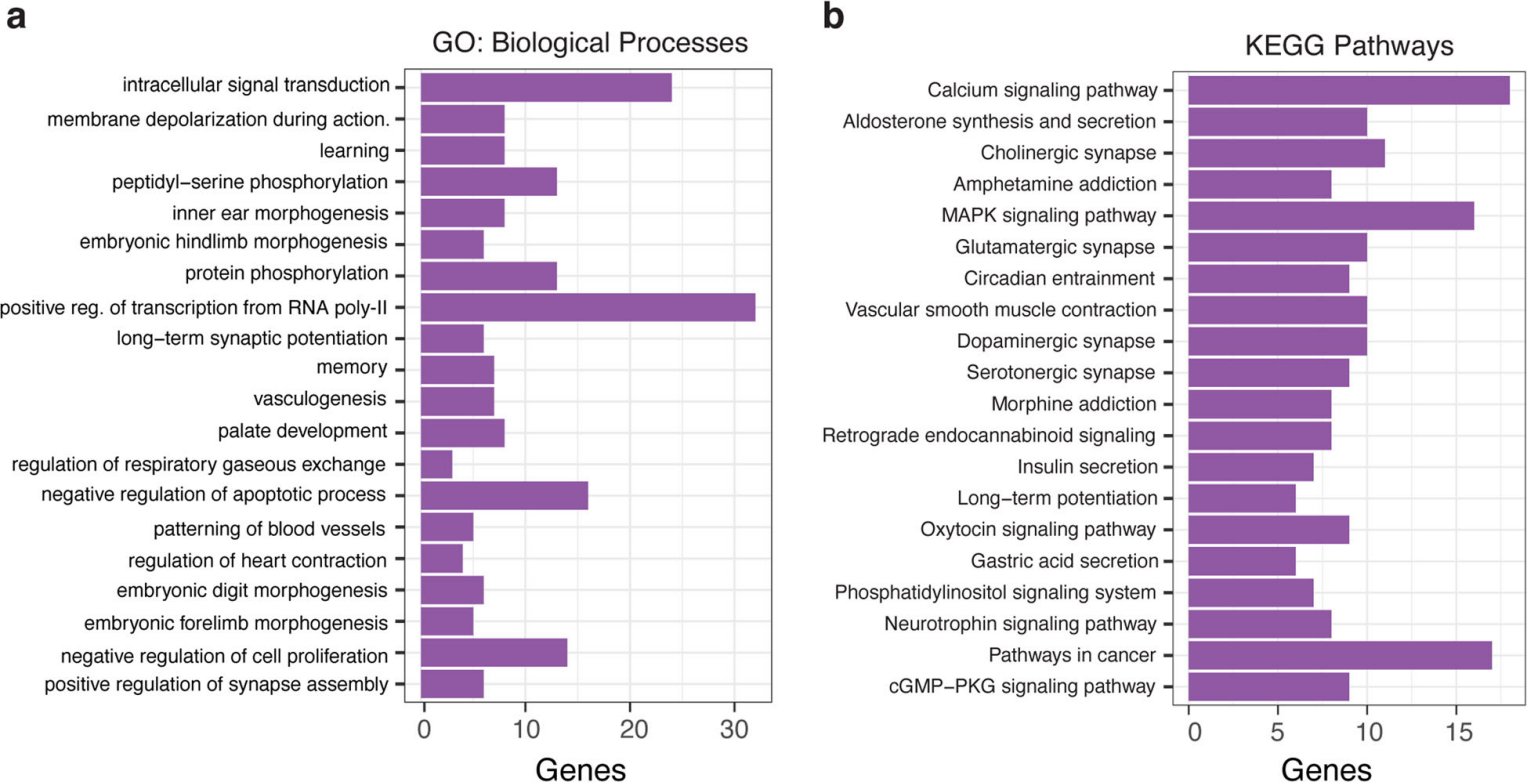
Gene Ontology (GO) and pathway analyses of genes located in the vicinity of significant differentially methylated regions (DMRs). Bar plots displaying enriched (**a**) biological processes GO terms and (**b**) KEGG pathways for DMR annotated genes. The plots show significantly enriched GO terms and pathways (*p* < 0.05)

In order to further characterize genes associated with DMRs, gene ontology and pathway analysis were carried out using an online platform DAVID [33, 34]. When using the 600 genes having one or more DMRs (Additional file 2: Table S4), GO analysis revealed significant overrepresentation of biological and molecular functions related mainly to signal transduction, cell proliferation, apoptotic process, vasculogenesis and embryo development (Fig. 4a, Additional file 2: Table S6). Among the molecular functions, DMRs were enriched in genes encoding proteins involved in calcium and zinc ion binding, voltage-gated calcium channel activity, ATP binding and transcription coactivator activity (Additional file 2: Table S7). Significant enrichment of several pathways was found using the KEGG database: notably, calcium-, MAPK-, vascular smooth muscle contraction-, Oxytocin- and cGMP-PKG signaling pathways (Fig. 4b). In addition, WikiPathways analysis revealed a network of genes known to be involved in multiple functions (Additional file 1: Figure S4, Additional file 2: Table S8). Notably among genes differentially methylated with one or multiple DMRs, several encode proteins involved in immune function and inflammatory processes (*HDAC4, AKT1,* and *IRAK1*), proliferation and apoptosis (WNT/β-catenin signaling *WNT7A*, *MAP3K6, BCL2)*, tissue remodeling (*ADAMTS2, ADAMTS14, ADAMTS17*) and the corticotropin releasing hormone signaling pathway, which relates to both trophoblast invasion (*TFAP2A*, hypomethylated DMR in exon 2) and angiogenesis (*PRKCA* and *PRKCG*, hypomethylated in intron 13 and hypermethylated in exon 5, respectively).

Pro-inflammatory mechanisms may be favored by epigenetic changes in the *HDAC4* gene (two hypomethylated DMRs in intron 1 and one hypermethylated DMR in intron 2) and hypermethylation of two DMRs associated to *AKT1* gene (in *AKT1* intron 1). In addition, the hypomethylation of *IRAK1* promoter and two hypomethylated DMRs on exon 1 and CpG island, may contribute to reinforce pro-inflammatory reactions through activation of TLR signaling. The *Wnt7A* gene, which is involved in proliferation, contains two hypomethylated DMRs in intron 3 and is over-expressed as shown from RNAseq data. We observed also the hypomethylation of one DMR in each of the promoters of *MAP3K6* and *BCL2* genes that regulates apoptosis. The methylation changes in *HDAC4* as reported above may affect also tissue remodeling as low expression has been associated with increased MMPs activity. This is consistent with the hypomethylation and increased expression of *ADAMTS17*.

## Discussion

Studies in both the human and bovine species have shown that the endometrial DNA methylome is highly dynamic and is submitted to changes throughout the oestrus cycle [35, 36], at time of early pregnancy [12] and relating to reproductive diseases [36, 37]. Several studies aimed at deciphering epigenetic changes in various types of cells following LPS challenge [27, 38, 39]. However, to our knowledge, this is the first attempt made to study genome-wide DNA methylation changes in a pure population of bovine endometrial epithelial cells (bEECs) exposed to LPS. Differentially methylated regions (DMRs) were identified here after controlling for the individual cow effects in the analysis and using combined results for 2 and 8 μg/ml LPS. In addition, for subsequent interpretation, only significant DMRs were kept which were found consistent between the 2 μg and 8 μg/ml dosages and in all three cows. The remaining significant DMRs were further scrutinized for changes reported in the literature especially for those relating to differential gene expression shown in our RNAseq study from the same cells [28].

In our dataset, LPS-treated groups expressed a significant trend for global DNA hypomethylation whereas, during the same period of time, the inverse trend was observed in controls. Despite differences in the LPS dosage and time of observation used, our results are consistent with previous studies in human and mouse showing that bacterial and viral infection induces hypomethylation of host cell DNA [40–42] and the decrease in methylation observed in other bovine cell types following LPS challenge [27, 38]. Globally, DNA methylation changes were enriched in sub-telomeric regions. This is in accordance with the fact that regions adjacent to telomere are rich in CpG islands [43, 44]. Although methylation changes induced by LPS occurred on all chromosomes they were more abundant on the X chromosome. Interestingly, the X chromosome had more hypomethylated genes compared with the autosomal chromosomes. Enrichment of DMRs and aberrant DNA hypomethylation of the X chromosome genes have also been reported in uterine leiomyoma [45] and in ovarian, cervical and breast cancers [46, 47].

The analysis of individual DMRs revealed that a number of them mapped to genes involved in the control of endometrial function and/or were related to endometrial dysfunction and infertility as documented mainly in humans and mice. The three main pathways associated specifically with the control of endometrial function, namely *i*) proliferation and differentiation, *ii*) cell migration, cell adhesion and extracellular matrix remodeling and *iii*) immune responses will be subsequently discussed especially in the light of corresponding changes in gene [28], protein expression [32], and phenotypic response to LPS [31] from the same cells.

### Cell proliferation and differentiation

Class II histone deacetylases (HDACs) are signal transducers often acting as co-repressors of transcription by removing histone acetylation [48], thereby influencing chromatin structure. The over-expression of HDACs such as HDAC4 has been associated to pathologies including cancer [49, 50]. HDACs can promote cell proliferation through repression of cyclin dependent kinase inhibitors such as p21 and simultaneous activation of CDK1 and CDK2 [51, 52]. The impacts of LPS on *HDAC4* methylation associated with a lower expression of several members of the HDAC’s family [28] on the proliferation of bovine endometrial cells would need specific investigations.

Wnt signaling pathway is also involved in cell proliferation and differentiation in the endometrium. Among genes from this family, *Wnt7A* encodes a key protein for the control of β-catenin and its increased expression is observed during the proliferative phase in human endometrial luminal epithelial cells [53]. Increased expression of these genes has been associated to proliferative activity of cancer cells [54] and resulted in endometrial dysfunction with altered uterine receptivity for embryo implantation [55, 56] which may result from deregulation of downstream genes important for endometrial function such as *FOXa2*, *LIF*, and *MSX1* [57].

Overall, epigenetic alterations corresponding to HDACs and WNT signaling are consistent with associated changes in gene expression induced by LPS. Further studies would be needed to demonstrate their specific role as part of the mechanisms explaining the strong proliferative phenotype observed in this model [31] and in different cell types [58].

### Cell migration, cell adhesion and extracellular matrix remodeling

Various effects of LPS on certain proteins from the ADAM’s family are the metalloproteases, which control fibrillary collagen processing and extracellular matrix organization. From our recent RNAseq results, the over-expression of *ADAMTS1* and *ADAMTS17* mRNAs were observed. Some of the roles ADAMTS1 on endometrial function have been described whereas less information exists for ADAMTS17. ADAMTS1 participates in the bovine endometrial remodeling at time of implantation and placental development [59], promotes epithelial cell invasion [60], and favors migration and alter adhesion [61, 62]. However, DNA methylation changes found here concerned *ADAMTS2, ADAMTS14* and *ADAMTS17*. The over-expression of *ADAMTS17*, has been associated to increased cell growth in cancer cells [63], and its hypo-methylation and over-expression from RNAseq [28] are consistent with the proliferative phenotype we observed and increased expression of proteins involved in tissue remodeling, and alterations of cell structure and cell adhesion pathways found in the same cells [32].

DNA methylation changes in genes from the corticotropin signaling network could be of biological significance due to the possible involvement of the proteins encoded by these genes in trophoblast invasion (*TFAP2A*) [63–65] and vascularization (*PRKCA* and *PRKCG*) [66–68]. Although interesting in view of future comparative studies, their role for tissue remodeling could be less critical in this model, as none of these genes were differentially expressed in response to LPS treatment [28].

### Inflammation and immune responses

Multiple signaling pathways are indeed activated following interaction between LPS and its most important receptor TLR4. This pleiotropic signaling response after activation of Toll-like receptors is likely critical to ensure efficient innate immune defenses against pathogenic bacteria. The underlying molecular mechanisms behind such sustained functional effects leads to changes in DNA methylation at regulatory regions of genes encoding proteins involved in such pathways. This change in chromatin structure in transcriptional regulatory regions results in the consequential changes of mRNA expression. Thus, LPS-TLR4 interactions leads to massive reprogramming of innate immune-mediated response pathways and our results reveal such changes at both the transcriptional and epigenetic levels measured at 24 h. Several studies have shown that class II HDACs such as HDAC4 are also key regulators of inflammatory response in immune cells with either pro- or anti-inflammatory roles [69–71]. Due to these roles, the functional significance of multiple differential methylation of this gene would deserve further studies.

AKT1 regulates negatively the immune response and specifically the production of IFNβ through the inhibition of both TLR-induced MyD88 phosphorylation and NF-kB/IRF3 signaling [72]. In contrast, Interleukin-1 receptor-associated kinases (IRAKs) are key proteins regulating positively both IL-1R- and TLR-mediated signaling [73]. As mentioned above, further work is needed to identify the specific steps and mechanisms involved in differential methylation observed here. However, it may be hypothesized from these results that in LPS-treated cells, the strong activation of the TLR-NF-kβ pathway, and the over expression of TNF receptor associated factors (TRAFs) acting as NF-kβ activators [28] may result from the hypermethylation of *AKT1,* which normally represses the above pathway and the hypomethylation of *IRAK1,* which activates TLR signaling.

We observed also a differential methylation of the peroxisome proliferator activated receptor alpha (*PPARA*, hypomethylation in intron 1), which could be of interest due to its possible role in enhancement of inflammation and restoration of physiological conditions in the endometrium following LPS challenge [74].

Despite a short time of exposure and the relatively low LPS dosage used in this study when compared to concentrations observed during natural or experimental infection [26, 29] a significant number of epigenetic changes which were related to genes involved in endometrial function were observed. It is possible that the epigenetic changes induced by LPS observed here could contribute to long term disturbances of gene expression and endometrial function which may not be favorable to tissue recovery and the establishment of pregnancy. Future studies, based especially on systems allowing long term cell culture and combining the different types of endometrial cells, would deserve further investigations to fully demonstrate the biological significance of these methylation changes in the context of disease.

## Conclusions

LPS induces changes in DNA methylation patterns of bovine endometrial epithelial cells, towards mainly hypomethylation that correlates with overall increased gene expression. LPS affects some specific genes and networks related to inflammation, cell proliferation, cell migration and tissue repair and vascularization. The possible impacts of these changes in methylation marks on long term alterations of endometrial receptivity and implantation would deserve further investigation.

## Methods

### Isolation of bovine endometrial epithelial cells

The uterine horns from three Swedish Red Breed cows were collected from slaughterhouse (Lövsta, Uppsala, Sweden) and were immediately transferred to the laboratory on ice. Cell isolation was performed within 1 h after slaughter as described previously [28]. Briefly, small pieces (2–3 mm) of bovine endometrial tissue were incubated for 2 h at 39 °C with 250 U/mL of collagenase IV (Sigma, St. Louis, MO, USA) and 250 U/mL of hyaluronidase (Sigma), in PBS containing 2% bovine serum albumin (BSA). The bEECs were separated from fibroblasts and blood cells by using a 40 μm cell strainer. Then bEECs were cultured at 39 °C, 5% CO_2_ with F-12 medium with 10% of fetal bovine serum (FBS, Gibco™ heat inactivated, virus and mycoplasma free, South America Origin). Sub-cultivations were performed when epithelial cells attained approximately 90% confluence. The purity of the epithelial cell culture was estimated by morphological observation and confirmed by anti-cytokeratin 18 antibody (Abcam, Cambridge, UK) and anti-vimentin V9 antibody (Abcam) immunofluorescence staining. Flow cytometer analysis further demonstrated a high degree of purity > 98% [31, 75]. Falcon® 25cm^2^ rectangular canted neck cell culture flask with vented cap were used for cell culture (Falcon, ref. 353,108). According to the manufacturer, the nonpyrogenic test was less than 0.1 EU/ mL.

### LPS challenge and genomic DNA isolation

According to the guide from Sigma, LPS (L2630-10MG O111:B4, Sigma-Aldrich) was reconstituted by 2 mL LAL reagent water (W50–640, Lonza, Walkersville, MD, USA) to a stock concentration of 5 mg/mL. In addition, according to the certificate of analysis from Sigma, the potency (Sample Endotoxin Unit, EU/mg) was ≥500,000 EU/mg. The reconstituted LPS was tested with PyroGene recombinant Factor C endotoxin detection system (Catalog No.: 50-658 U, Lonza) in our lab, which showed ≥600,000 EU/mg. At passage 5, in vitro cultured bEECs were either unexposed (control) or exposed to 2 or 8 μg/mL *E. coli* LPS (O111:B4; Sigma). These concentrations of LPS, which may mimic those during days after acute infection, are in the lower range of those previously reported in cow uterine fluid in case of clinical endometritis and/or in vivo experimental infection [29, 30]. They were also chosen here, based on our previous experiments showing effects of LPS on cell survival and proliferation profiles and proteomic profiles [31, 32] and the same biological material was used (same cells exposed to same LPS dosages and time point) as in our former RNAseq study [28]. The bEECs were collected at time 0 h (before LPS challenge) and 24 h after challenge as in [27, 28], by using TrypLE™ express (Gibco-BRL 12605) and washed twice with Dulbecco’s PBS (DPBS; Life Technologies Inc. Gibco-BRL, Grand Island, NY, USA). Approximately two million cells were obtained from each treatment and kept at − 80 °C. Genomic DNA was extracted by using the Allprep DNA/RNA/miRNA Universal Kit (Qiagen, Hilden, Germany). Each prepared DNA sample was tested by nanodrop to determine the purity, quality and quantity. For all samples, the ratios of the absorbance at 260 nm and 280 nm (A260/280) and at 260 nm and 230 nm (A260/230) were between 1.8–2.0 and 2.1–2.4, respectively.

### Library preparation and sequencing

Libraries for RRBS were generated by *Msp*I digestion of the DNA followed by end-repair/A-tailing and 5mC adaptor ligation; bisulfite conversion; and subsequent PCRs. Libraries were sequenced at The Babraham Institute, UK using Illumina HiSeq 2500 which generated 17–21 million 50 bp single-end reads per sample.

### Read mapping and identification of DMRs

Raw reads were trimmed for low quality sequences, and adapter sequences were removed using Trim Galore [76]. Filtered reads were then aligned to the bovine reference genome sequence (bosTau8 UMD3.1) using BS-Seeker2 [77]. Only the cytosines with coverage of at least five reads detected in all three cows were used for differential methylation analysis, which was performed with the R package, methylKit v1.8.1 [78]. Logistic regression model was used to separate the effect of individual variation and other covariates from the treatment effect. Since the control and treatment were paired samples, the full model (~ treatment + cow) was used to control for the individual cow effect from the treatment effect. The genome was tiled for 100 bp in order to find differentially methylated regions (DMRs). DMRs with at least two CpG sites with a methylation difference ≥ 10% and *q*-value < 0.05 between sample groups were considered for further analysis. Ensembl gene annotation version 84 was used in the methylation analysis of genic regions, whereas CpG islands and repetitive element annotations for bosTau8 genome were obtained from the UCSC genome browser database (https://genome.ucsc.edu/).

### Gene ontology over-representation and pathway analyses

Gene ontology (GO) overrepresentation analysis of DMR-associated genes was performed using DAVID functional annotation tool [33, 34]. All annotated genes in *Bos taurus* genome were used as background for GO analysis. Pathway enrichment analysis was performed using the KEGG database available within DAVID platform, and with WikiPathways database (https://www.wikipathways.org/).

## Supplementary information

**Additional file 1: Figure S1.** Cell culture protocol of bEECs. On passage 5, DNA from bottle ‘A’ was extracted at time 0 h. At this time bottles ‘B’, ‘C’ and ‘D’ were treated with 0, 2 and 8 μg/mL of LPS, respectively. After 24 h, DNA was extracted separately from bottles ‘B’, ‘C’ and ‘D’. **Figure S2.** Distribution of CpG coverage in bEECs obtained from RRBS. **Figure S3.** Correlation of CpG methylation levels between control 0 h and 24 h, 2 μg/mL, 8 μg/mL bEECs samples. **Figure S4.** Network plot of genes showing significantly over-represented pathways from the WikiPathways database. Genes present in multiple pathways are highlighted in red. Critical genes for endometrial function are highlighted in green boxes. The plot shows significantly enriched pathways (*p* < 0.05).

**Additional file 2: Table S1.** Sequencing and mapping statistics of RRBS data. **Table S2.** Significant differentially methylated regions in bEECs 24 h control group compared to 0 h group. **Table S3.** Significant differentially methylated regions in LPS treated bEECs compared to 24 h control. **Table S4.** Genomic distribution of DMRs in LPS treated bEECs and annotated genes in the vicinity. **Table S5.** DMRs negatively correlated with gene expression data in LPS treated bEECs. **Table S6.** GO terms enrichment analysis for Biological Processes of genes associated with significant DMRs. **Table S7.** GO terms enrichment analysis for Molecular Functions of genes associated with significant DMRs. **Table S8.** Pathways analysis of genes associated with significant DMRs.

## Data Availability

All RRBS raw sequencing data were deposited in the European Nucleotide Archive (ENA) under accession number PRJEB36023. Raw RNAseq sequencing data are also available in the ENA database under accession PRJEB34011. All reference data including reference genome sequence (Accession ID: GCA_000003055.4), CpG islands and repetitive element annotations for bosTau8 are available in the UCSC genome browser database (https://genome.ucsc.edu/). Gene annotations (Accession ID: GCA_000003055.3) are available at Ensembl genome database https://www.ensembl.org/.

## Abbreviations

RRBS: Reduced Representation Bisulfite Sequencing
LPS: Lipopolysaccharide
bEECs: bovine endometrial epithelial cells
DMRs: Differentially Methylated Regions
*E. coli*: *Escherichia coli*
bosTau8: *Bos taurus* 8
GO: Gene ontology
KEGG: Kyoto Encyclopedia of Genes and Genomes
RNAseq: RNA sequencing
mRNA: messenger RNA
ENA: European Nucleotide Archive
