# Supplementary material for: LPS-treatment of bovine endometrial epithelial cells causes differential DNA methylation of genes associated with inflammation and endometrial function
Source: BMC Genomics. 2020 Jun 3;21:385. doi: 10.1186/s12864-020-06777-7 (PMC7268755; doi:10.1186/s12864-020-06777-7)
Supplement: Supplementary file 1 — Additional file 1: Figure S1. Cell culture protocol of bEECs. On passage 5, DNA from bottle ‘A’ was extracted at time 0 h. At this time bottles ‘B’, ‘C’ and ‘D’ were treated with 0, 2 and 8 μg/mL of LPS, respectively. After 24 h, DNA was extracted separately from bottles ‘B’, ‘C’ and ‘D’. Figure S2. Distribution of CpG coverage in bEECs obtained from RRBS. Figure S3. Correlation of CpG methylation levels between control 0 h and 24 h, 2 μg/mL, 8 μg/mL bEECs samples. Figure S4. Network plot of genes showing significantly over-represented pathways from the WikiPathways database. Genes present in multiple pathways are highlighted in red. Critical genes for endometrial function are highlighted in green boxes. The plot shows significantly enriched pathways (p < 0.05). [file 12864_2020_6777_MOESM1_ESM.docx]

**Additional file 1: Supplementary Figures**


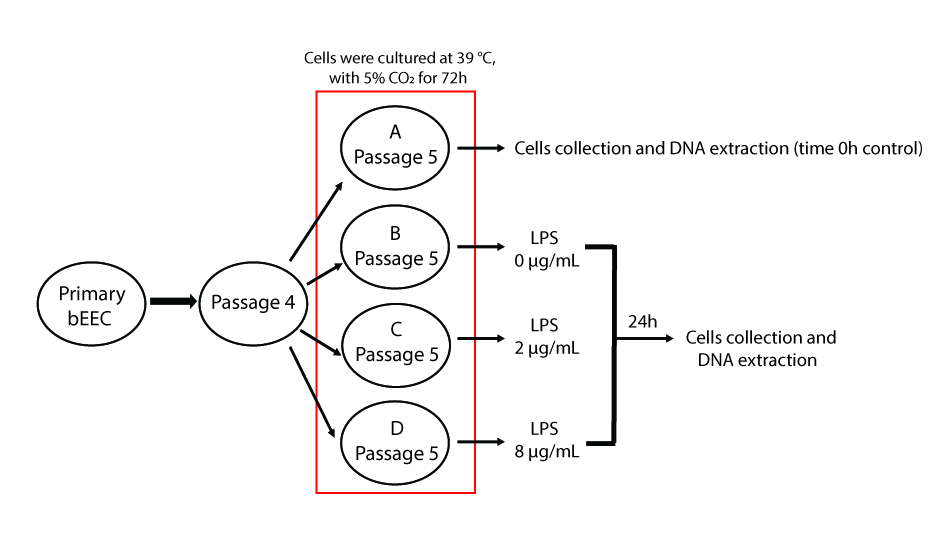


**Figure S1.** Cell culture protocol of bEECs. On passage 5, DNA from bottle ‘A’ was extracted at time 0 h. At this time bottles ‘B’, ‘C’ and ‘D’ were treated with 0, 2 and 8 µg/mL of LPS, respectively. After 24 h, DNA was extracted separately from bottles ‘B’, ‘C’ and ‘D’.

**Figure S2.** Distribution of CpG coverage in bEECs obtained from RRBS.

**
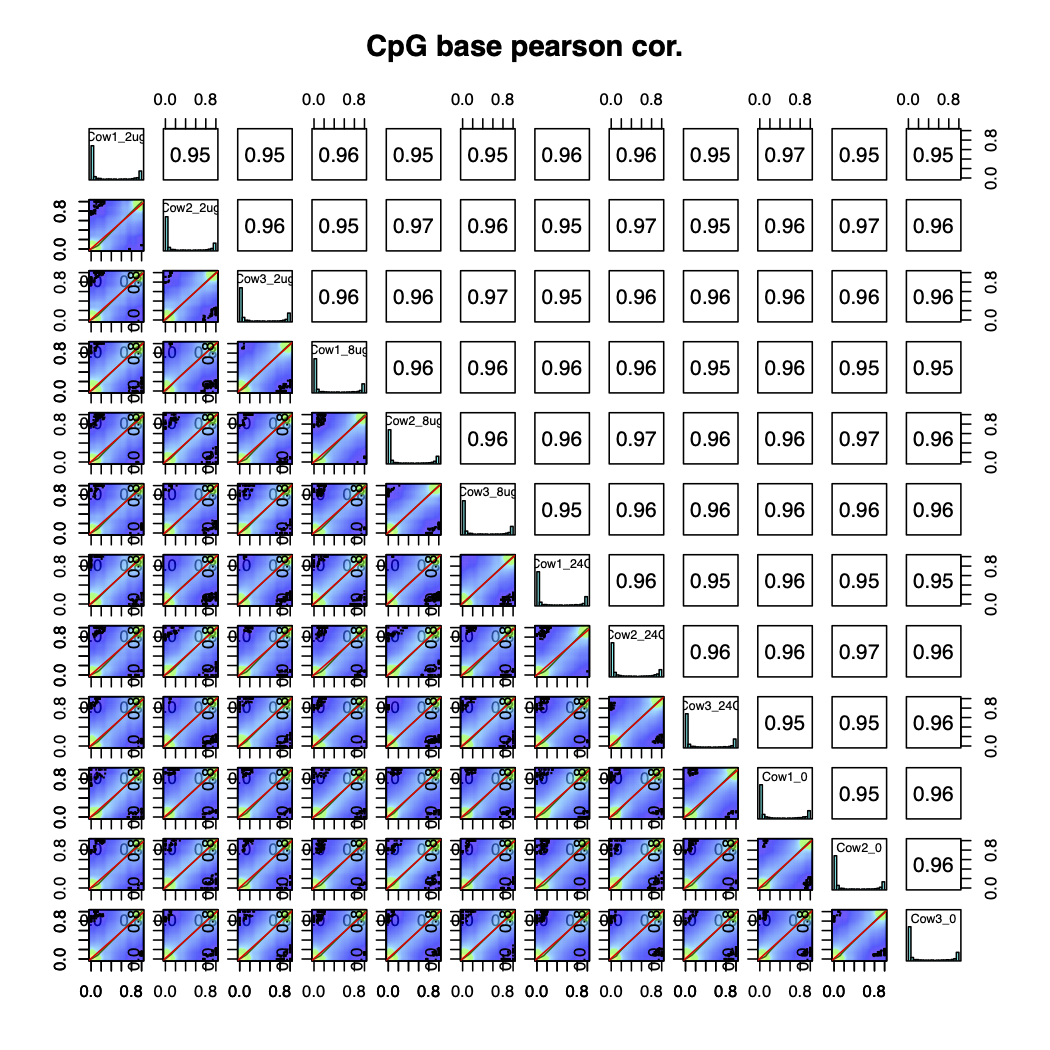
**

**Figure S3.** Correlation of CpG methylation levels between control 0 h and 24 h, 2 μg/mL, 8 μg/mL bEECs samples.


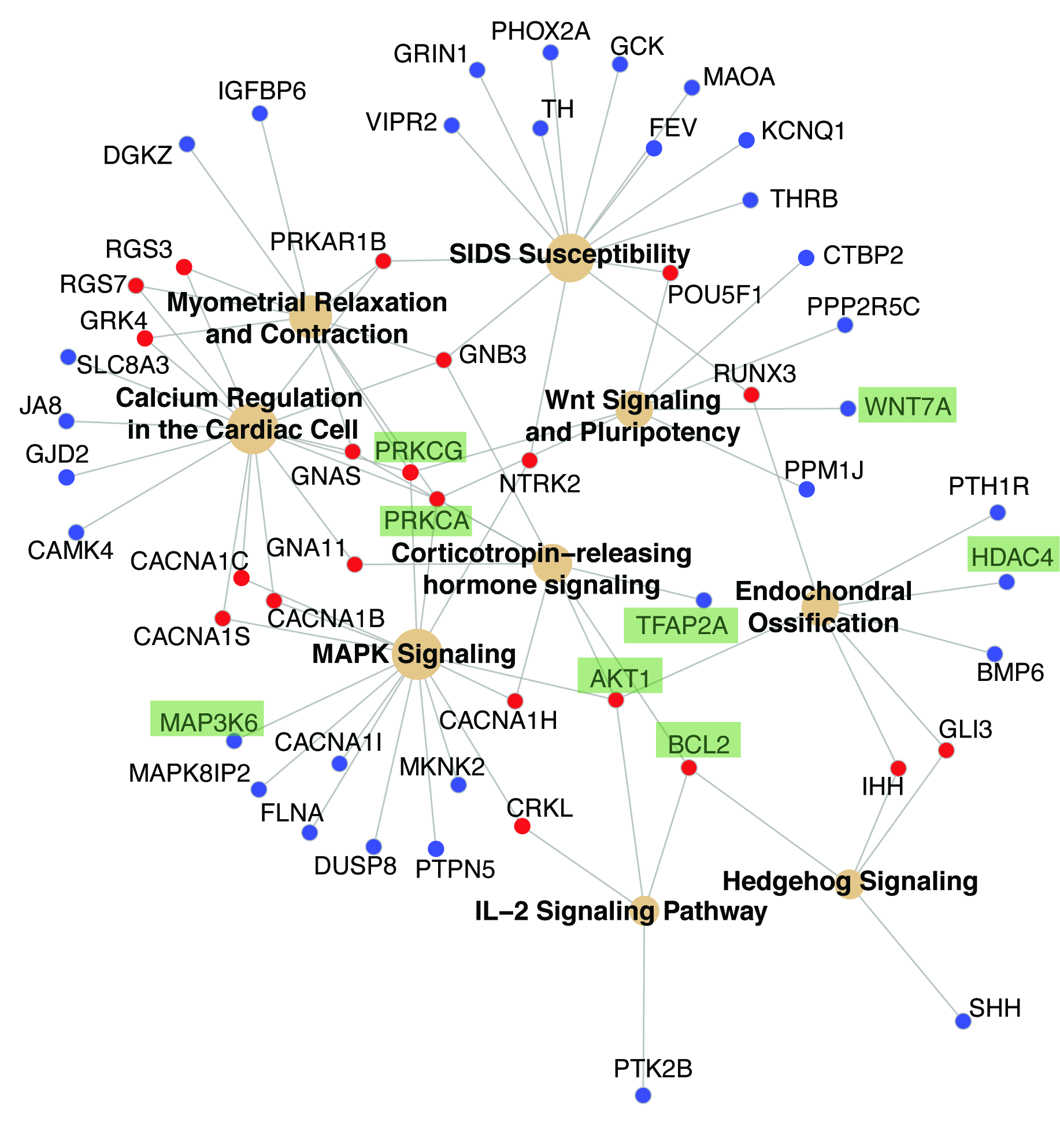


**Figure S4.** Network plot of genes showing significantly over-represented pathways from the WikiPathways database. Genes present in multiple pathways are highlighted in red. Critical genes for endometrial function are highlighted in green boxes. The plot shows significantly enriched pathways (*p* < 0.05).
